# BSR 2020 Annual Meeting: Program

**DOI:** 10.5334/jbsr.2311

**Published:** 2020-11-13

**Authors:** Anne-Sophie Vanhoenacker, Flavien Grandjean, Van Hoe Lieven, Annemie Snoeckx, Piet Vanhoenacker, Raymond Oyen

**Affiliations:** 1 University Hospitals Gasthuisberg, Leuven, BE; 2 CHC Liège MontLegia, BE; 3 OLV Ziekenhuis Aalst-Asse-Ninove, BE; 4 UZ Antwerpen, BE

**Keywords:** Annual Symposium, BSR, 2020

## Abstract

Different times call for different measures. The COVID-19 pandemic has forced us to search for alternative methods to provide an annual meeting which is equally interesting and has quality. For the Belgian Society of Radiology (BSR) 2020 Annual Meeting, the sections on Abdominal Imaging, Thoracic Imaging and the Young Radiologist Section (YRS) joined forces to organize a meeting which is quite different from the ones we have organised in the past. We have chosen to create a compact – approximately 5 hour – and entirely virtual meeting with the possibility of live interaction with the speakers during the question and answer sessions. The meeting kicks off with a message from the BSR president about radiology in 2020, followed by three abdominal talks. The second session combines an abdominal talk with COVID-related talks. We have chosen to include not only thoracic findings in COVID-19, but to take it further and discuss neurological patterns, long-term clinical findings and the progress in artificial intelligence in COVID-19. Lastly, the annual meeting closes off with a short movie about the (re)discovery of Röntgens X-ray, presented to us by the Belgian Museum for Radiology, Military Hospital, Brussels.

Different times call for different measures. The COVID-19 pandemic has forced us to search for alternative methods to provide an equally qualitative and interesting annual meeting. For the Belgian Society of Radiology (BSR) 2020 Annual Meeting, the sections on Abdominal Imaging, Thoracic Imaging, and the Young Radiologist Section (YRS) joined forces to organize a meeting that is quite different from the ones we have organized in the past. We have chosen to create a compact – approximately 5 hours – and entirely virtual meeting with the possibility of live interaction with the speakers during the question-and-answer sessions. The meeting kicks off with a message from the BSR president about radiology in 2020, followed by three abdominal talks. The second session combines an abdominal talk with COVID-related talks. We have chosen to include not only thoracic findings in COVID-19, but to take it further and discuss neurological patterns, long-term clinical findings, and the progress in artificial intelligence in COVID-19. Lastly, the annual meeting closes off with a short movie about the (re)discovery of Röntgens X-ray, presented to us by the Belgian Museum for Radiology, Military Hospital, Brussels.

The meeting will take place on **Saturday, November 14th 2020**.

The meeting starts at **9:00 AM** with a message from Dr. Piet Vanhoenacker (OLV Ziekenhuis Aalst-Asse-Ninove) (Picture 1), president of the BSR, with an overview of the activities of the BSR during the last year and the challenges that lie ahead.

The **first session (10:50–12:00)** focuses on abdominal imaging and is moderated by Dr. Annemie Snoeckx (Picture 12) (UZ Antwerpen) and Dr. Anne-Sophie Vanhoenacker (Picture 13) (YRS, University Hospitals Leuven). It contains three lectures about different subjects. First, Prof. Dr. Robert Dondelinger (Department of Radiology, University Hospital Sart Tilman, Liège) (Picture 2) will talk about biliary pathologies and the basic principles of treatment of bile duct obstruction. Next, Prof. Dr. Elleke Dresen (Picture 3), who is a staff member at UZ Gasthuisberg, Leuven and obtained a PhD dissertation on the multidisciplinary approach to locally advanced and recurrent rectal cancer, will give us a case-based approach as how to interpret Magnetic Resonance Imaging (MRI) of the rectum in this ‘How I do it’ talk. Finally, Dr. Philip Van Hover (Picture 4), staff member at the OLV Ziekenhuis Aalst-Asse-Ninove, with a special interest in uroradiology, will teach us tips and tricks in imaging and management of adrenal incidentaloma.

The **second session (11:00–12:30)** is a mix of abdominal imaging and COVID-related talks and is moderated by Dr. Lieven Van Hoe (Picture 14) (OLV Ziekenhuis Aalst-Asse-Ninove) and Dr. Flavien Grandjean (Picture 15) (YRS, CHC Liège MontLegia). First, Prof. Dr. Paul Meunier (Picture 5), Head of Radiology Department of CHU de Liège, will give an elaborate talk on the different types of abdominal internal hernias on the basis of their appearance via a normal or abnormal orifice. Next, Prof. Dr. Walter De Wever (Picture 6), thoracic radiologist and staff member at University Hospitals Leuven, will talk about the latest research on long-term pulmonary changes after COVID-19. Finally, Prof. Dr. Natalie Lorent (Picture 7), respiratory physician and staff member at the University Hospitals Leuven, will present the latest data on recovery after COVID-19 based on literature data as well as follow-up findings in UZ Leuven.

The **third and final session (12:20–13:25)** is moderated by Dr. Flavien Grandjean and Anne-Sophie Vanhoenacker. Prof. Dr. Sofie Van Cauter (Picture 8), certified neuroradiologist in ZOL Genk en UZLeuven, will discuss the various neurological and neuropsychiatric complications related to COVID-19. Next, the latest progress in artificial intelligence in COVID-19 will be discussed by Dr. Laurens Topff (Picture 9), radiologist at the Netherlands Cancer Institute – Antoni van Leeuwenhoek in Amsterdam, and Dr. Erik Ranschaert (Picture 10), radiologist at the ETZ Hospital in Tilburg, the Netherlands and artificial intelligence (AI) project manager. The annual meeting will end with a short movie on the (re)discovery of Röntgens X-ray, presented by René Van Tiggelen (Picture 11), radiologist and current Curator of the Belgian Museum for Radiology, Military Hospital, Brussels.

## Speakers

### Prof. Dr. Robert Ferdinand Dondelinger

**Figure:**
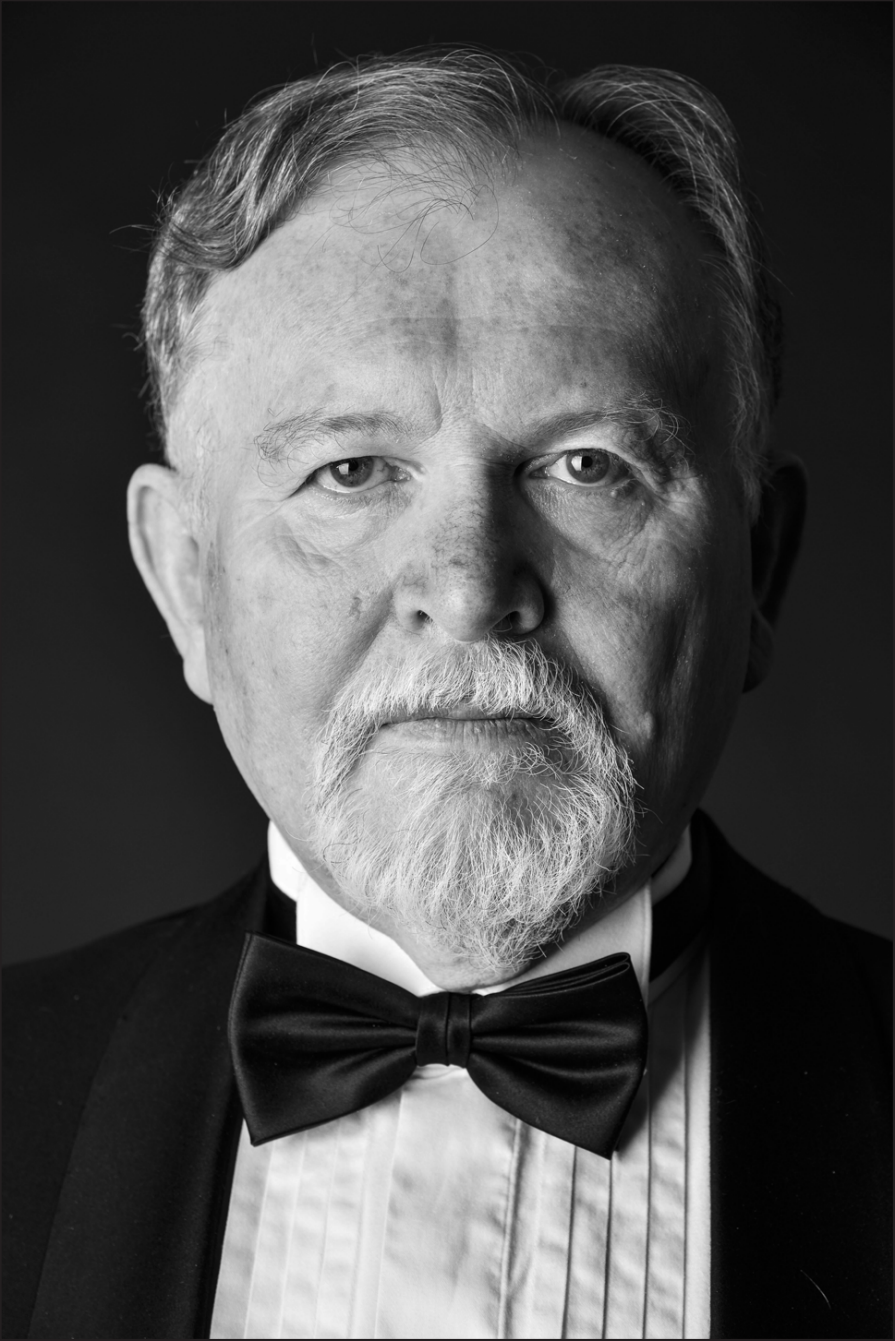


Prof. Dr. Robert F. Dondelinger (MD, PhD, Hon.FRCR (London)), graduated from University of Montpellier, France. After accomplishment of medical studies and residency training in radiology, he defended an inaugural doctorate thesis in France on ‘Abdominal Computed Tomography’ in 1978, earning the title of laureate of the Faculty of Medicine. During the 1980s, being in charge of visceral imaging at Centre Hospitalier of Luxembourg, Grand-Duchy of Luxembourg, he pioneered radiological interventions and authored articles and books in interventional techniques. In 1991, Robert F. Dondelinger was nominated full professor of Radiology at University of Liège, Belgium, and head of the Department of Medical Imaging of University Hospital Sart Tilman. He continued clinical research in vascular and visceral interventions and committed himself to international teaching. He produced many articles, contributed and edited books from 1991 to 2009, promoting imaging guided interventions. Professor Dondelinger was a member of European College of Angiography (ECA), founding member, secretary, and treasurer of the Cardiovascular and Interventional Society of Europe (CIRSE), founding president of the European Society of Thoracic Imaging (ESTI), founding member, president and gold medallist of the European Society of Gastrointestinal and Abdominal Radiology (ESGAR), president of the Royal Belgian Society of Gastroenterology (RGSGE), president of the Royal Belgian Society of Radiology (SRBR) and honorary member of other national or international scientific societies. At present, he is a consultant at the Department of Medical Imaging of University Hospital Sart Tilman, Liège.

### Prof. Dr. Elleke Dresen

**Figure:**
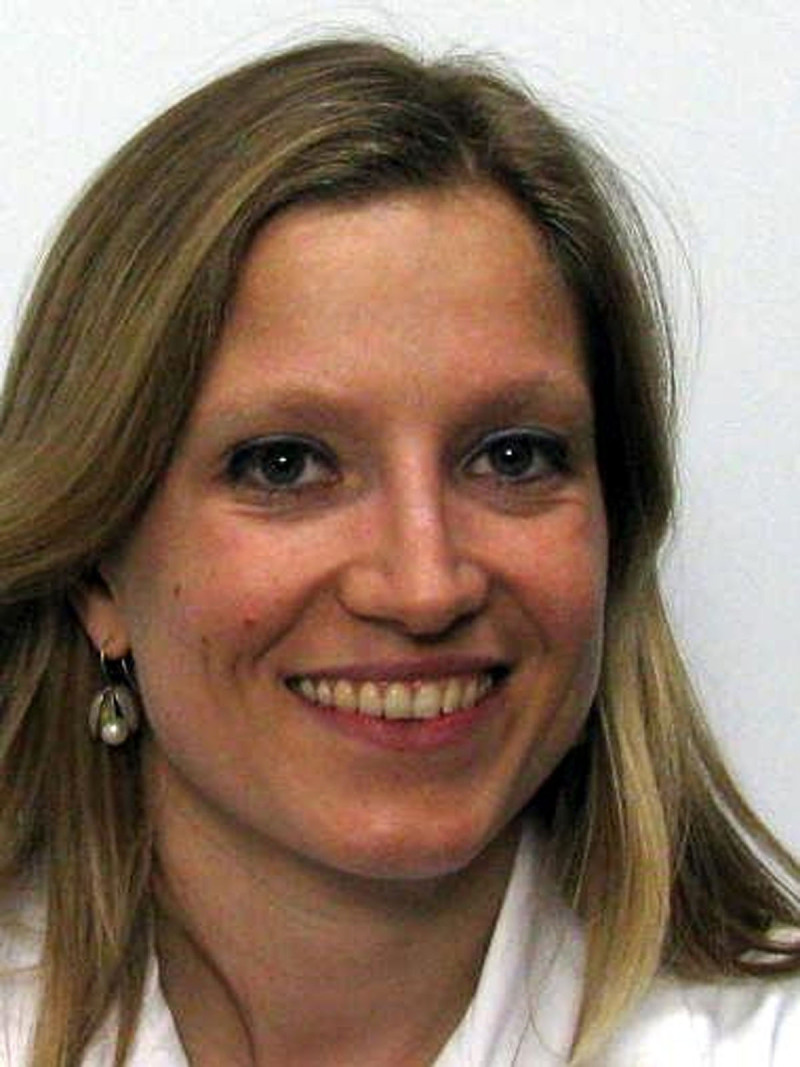


Prof. Dr. Raphaëla (Elleke) Dresen (MD, PhD) works as an abdominal radiologist at the University Hospitals Leuven since 2014. She obtained her medical degree from the University of Maastricht, the Netherlands, in 2006 and earned her PhD at the Faculty of Health, Medicine and Life Sciences in Maastricht in 2009. Her dissertation was addressed to the multidisciplinary approach to locally advanced and recurrent rectal cancer. She practices all aspects of abdominal radiology, including magnetic resonance imaging (MRI), computed tomography (CT), positon emission tomography coupled with CT (PET/CT), ultrasound, fluoroscopy and nonvascular abdominal interventions. She is an active member of the oncological multidisciplinary team meetings, especially concerning gastro-intestinal, colorectal, and gynaecological cancer patients. Since October 2017 she is partly employed as an assistant professor at KU Leuven. She is involved in the education of students, interns and residents. Her current research addresses oncological imaging, with a special focus on rectal cancer imaging and whole-body diffusion-weighted magnetic resonance imaging in patients with gastro-intestinal, colorectal, and gynaecological cancer with the aim of optimization of treatment planning and assessment of treatment response. She is a faculty member of the biannual ESGAR workshop “MR imaging of rectal cancer”.

### Dr. Philip Van Hover

**Figure:**
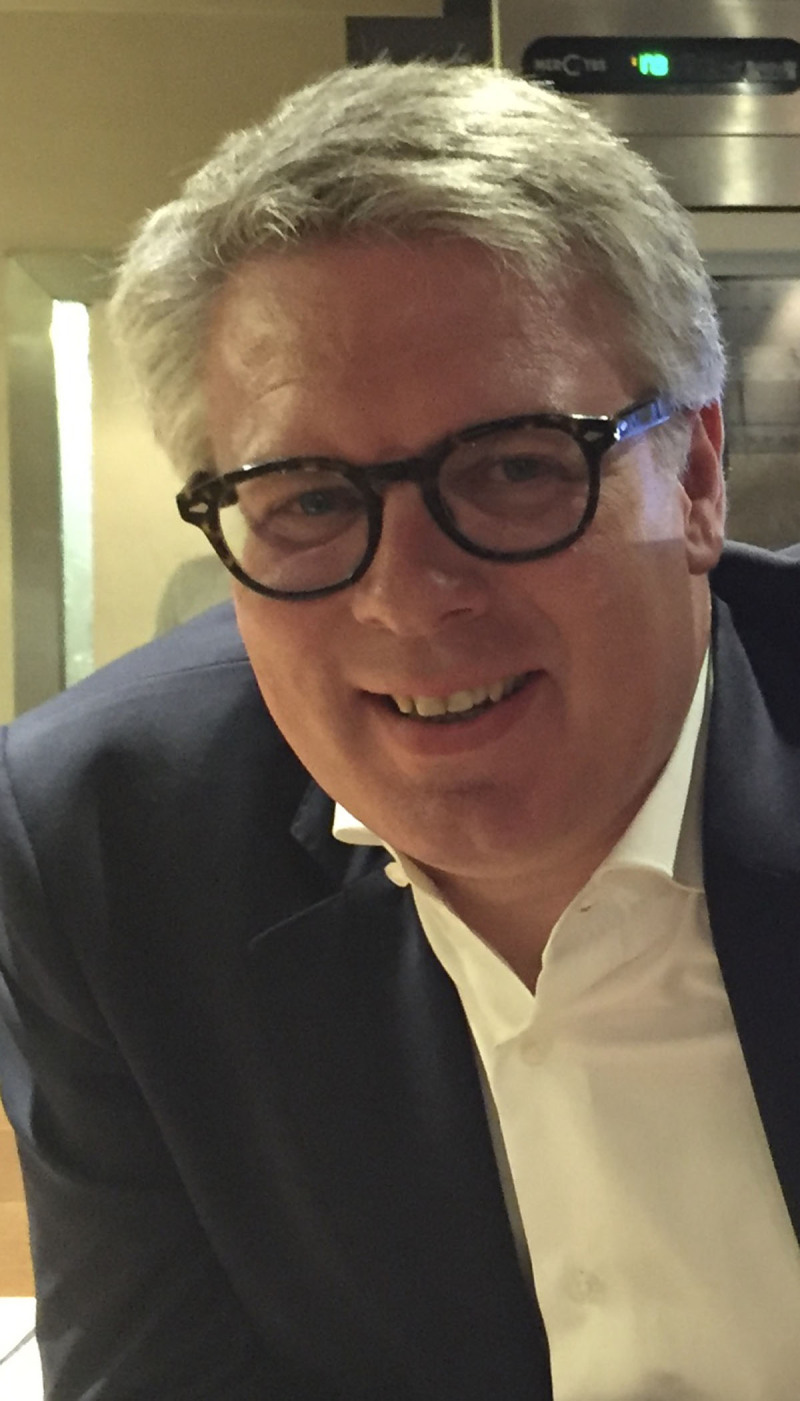


Born in 1967, Dr. Philip Van Hover (MD) did his medical studies at the Leuven University from 1985 until 1992. He trained in the department of radiology of Prof. A.L. Baert from 1992 until 1997. He was trained as a general radiologist with a special interest in uroradiology, thanks to Prof. R. Oyen. In 1997 he started as a general radiologist in the Onze Lieve Vrouw Ziekenhuis in Geraardsbergen, but in 2001 he changed to the Onze Lieve Vrouw Ziekenhuis in Aalst-Asse-Ninove, where he is still an active staff member of the department. His interests are in uroradiology, abdominal radiology, and interventional radiology. From 2013 until 2016 he was consulting interventional radiologist in the VINRAD department of Prof. L. Defreyne at the University Hospital in Gent.

### Prof. Dr. Paul Meunier

**Figure:**
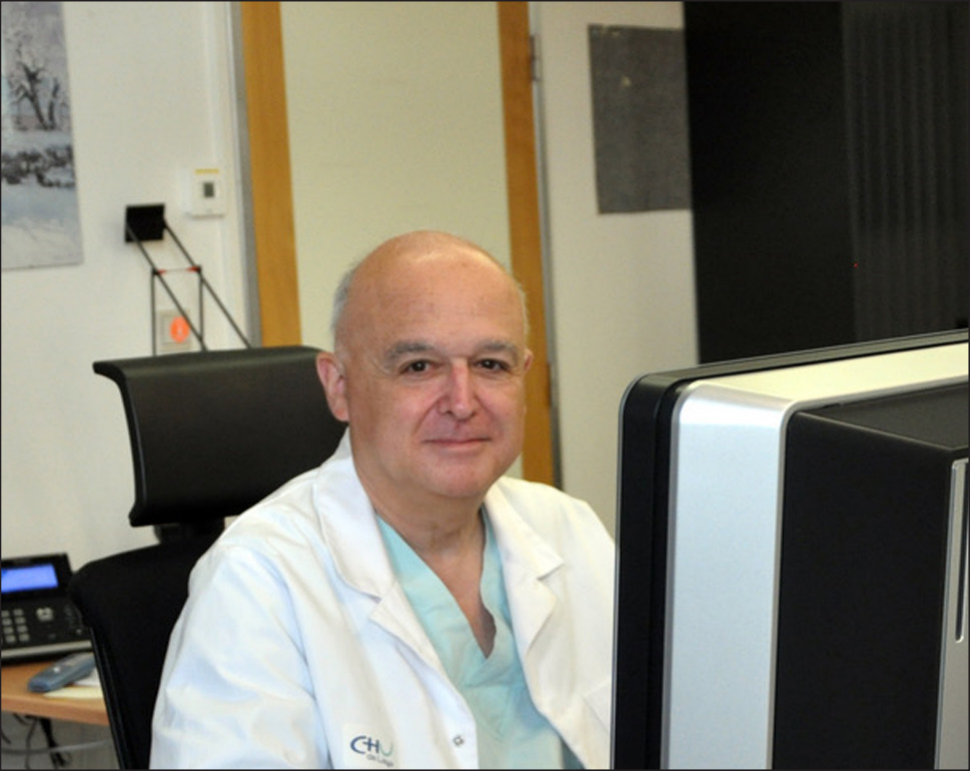


Prof. Dr. Paul Meunier (MD, PhD) is the Head of Radiology Department of CHU de Liège. He is a lecturer in medical imaging for the University of Liege and also a specialist in abdominal imaging. He has actively participated in several trials in collaboration with different departments (Urology, Gastroenterology, Nephrology, Pneumology, etc.) and done research work based on Crohn’s disease, which is one of his specialties. He received a Belgian Week of Gastroenterology award for the best research paper published in 2015. Dr. Meunier has been an author or co-author of around 20 international peer-reviewed publications. He is a member of international societies (French Society of Radiology, Radiological Society of North America, European Society of Gastrointestinal and Abdominal Radiology, Belgian Society of Radiology, etc.) and was President of the Belgian Society of Gastroenterology in 2017.

### Prof. Dr. Walter De Wever

**Figure:**
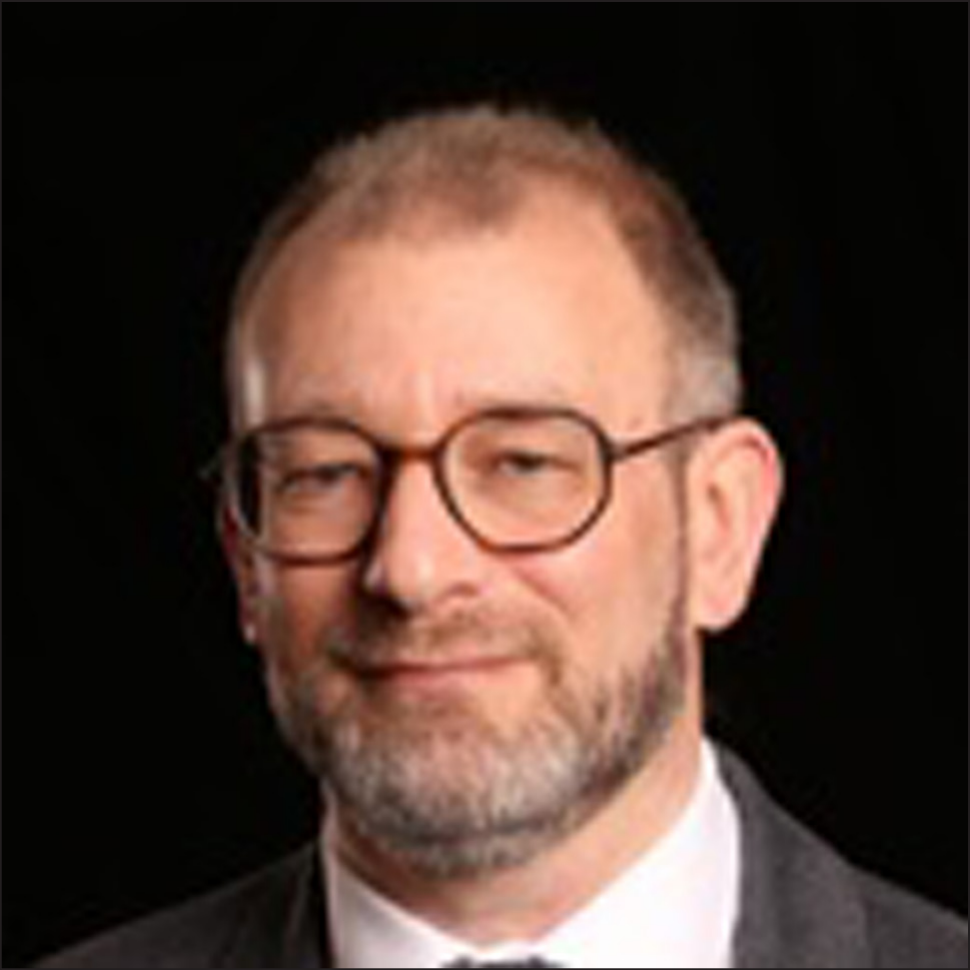


Prof. Dr. Walter De Wever (MD, PhD) is a radiologist specializing in thoracic radiology at the University Hospitals Leuven since 1997. He earned a PhD on “The Role of Integrated PET/CT in the Staging of Non-Small Cell Lung Cancer” in 2008. He was a member of the European Respiratory Society (ERS) since 2005 and chairman from 2006 until 2009. From 2009 until 2012, he was secretary of the imaging group of the ERS. From 2014 until 2018 he was chairman of the chest imaging group of the Belgian Radiological Society.

### Prof. Dr. Nathalie Lorent

**Figure:**
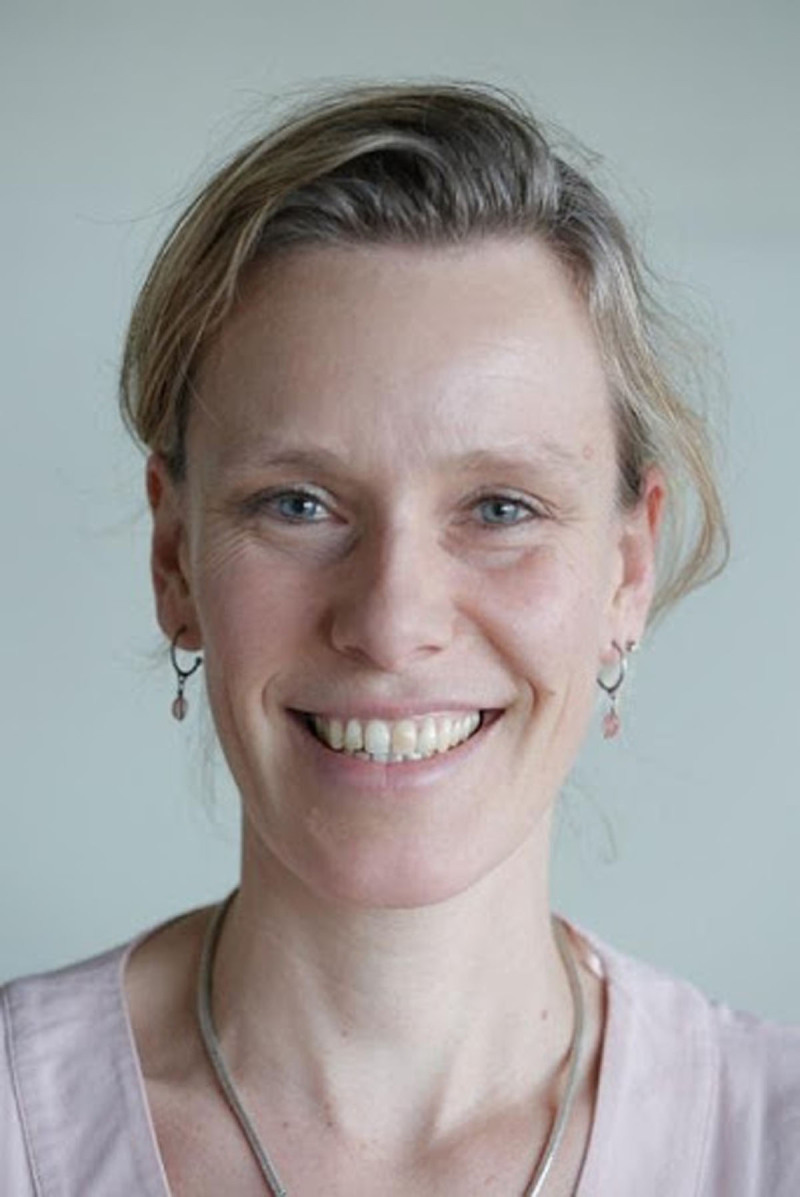


Prof. Dr. Natalie Lorent (MD, PhD) is a respiratory physician working in the University Hospitals Leuven since 2015. As a clinician, she mainly cares for patients with complex respiratory infections, bronchiectasis, and cystic fibrosis. Her major research interests are pulmonary mycobacterial diseases, including tuberculosis and non-tuberculous mycobacterial disease. Her teaching commitments include undergraduate, postgraduate, and clinical teaching.

Natalie studied medicine at the Catholic University of Leuven and carried out her internal and respiratory medicine training in both Belgium and the UK. Before clinical academia, she was a medical preceptor in the national HIV-programme‘s ART-roll-out in Botswana and worked for three years as a respiratory consultant in the University Hospital of Kigali in Rwanda. Subsequently, she started as a researcher at the Institute of Tropical Medicine in Antwerp, completing her PhD after four years working in Cambodia, before moving back to her hometown.

### Prof. Dr. Sofie Van Cauter

**Figure:**
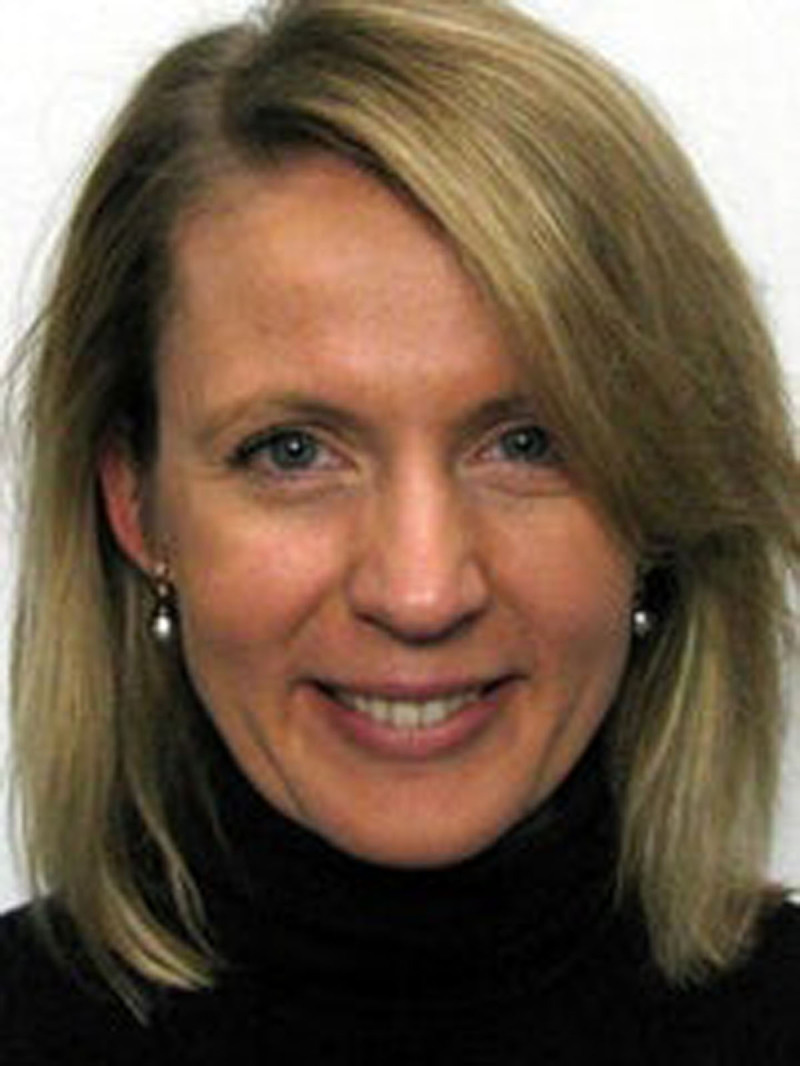


Prof. Dr. Sofie Van Cauter (MD, PhD) is a certified neuroradiologist in ZOL Genk and UZLeuven. She obtained a PhD in neuro-oncology on advanced imaging techniques in gliomas in the Catholic University of Leuven in 2013, awarded with the “wetenschappelijke prijs Prof. Dr. A. Baert”. She has special interest in the impact of artificial intelligence on neuroradiology and neuro-oncological, skull base, and neurovascular imaging.

### Dr. Laurens Topff

**Figure:**
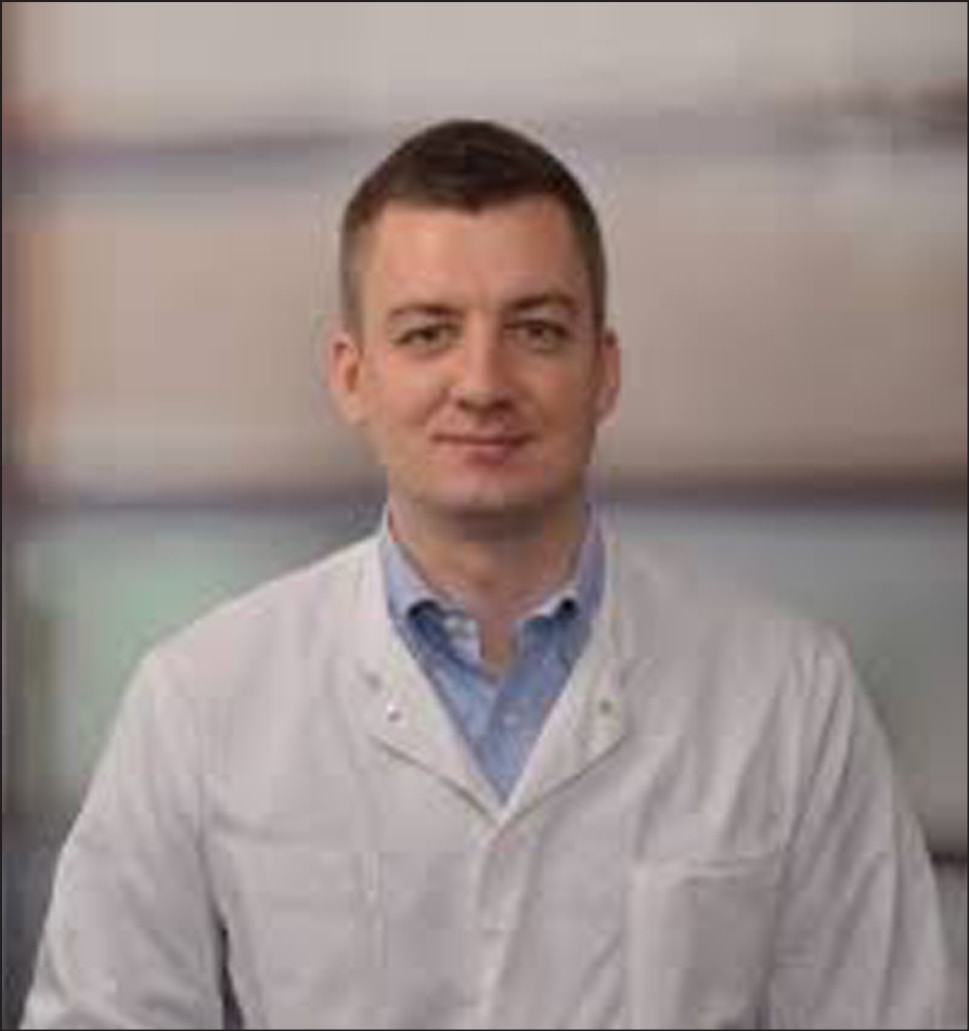


Dr. Laurens Topff (MD) is a radiologist at the Netherlands Cancer Institute – Antoni van Leeuwenhoek in Amsterdam. He is specialized in neuroradiology, head and neck radiology, and oncologic imaging. He received his Master of Medicine at KU Leuven. His specialist training was conducted at University Hospitals Leuven and ZOL Genk. He is a certified imaging informatics professional. He is currently involved in several projects that focus on implementation of artificial intelligence in radiology practice.

### Prof. Dr. Erik Ranschaert

**Figure:**
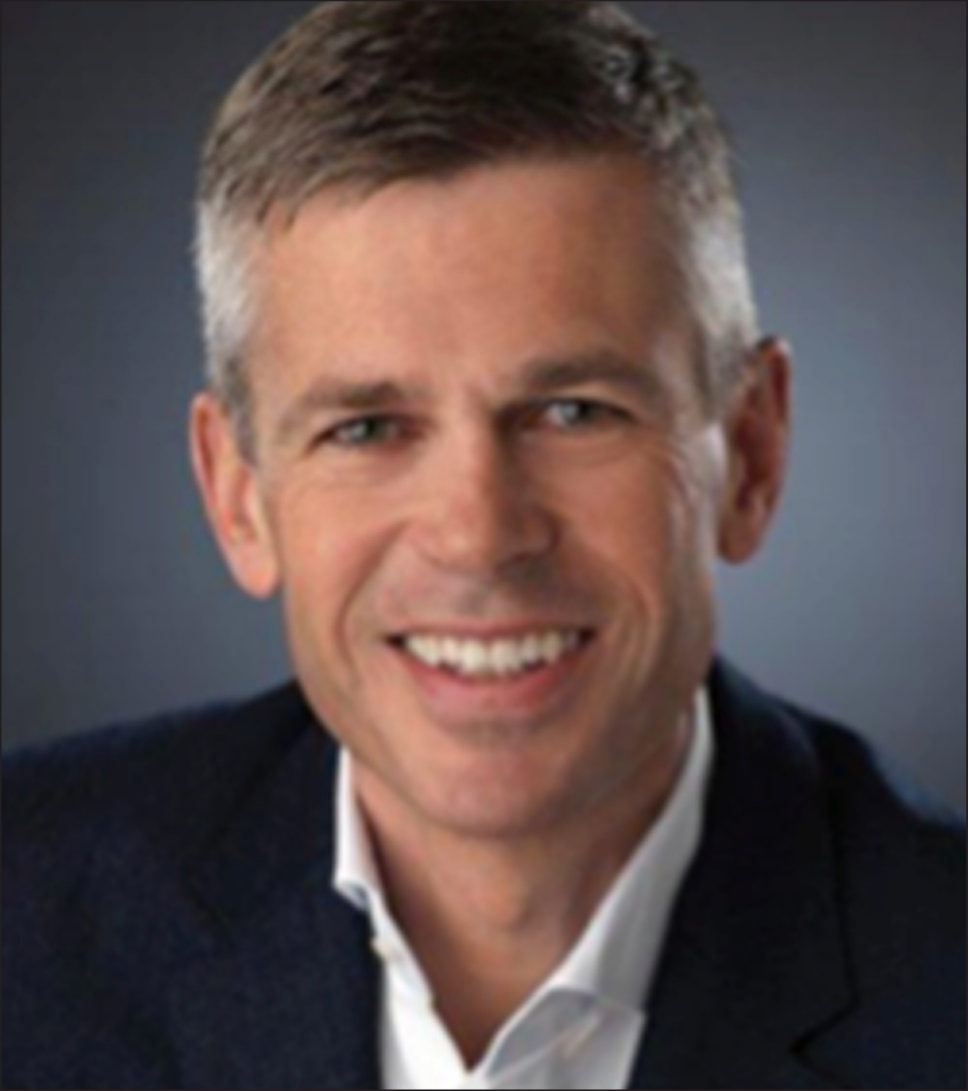


Prof. Dr. Erik Ranschaert (MD, PhD) studied medicine and radiology at the KU Leuven, Belgium. In 2016 he obtained his PhD degree at the University of Antwerp. He currently works as radiologist at the ETZ Hospital in Tilburg, the Netherlands. From his function as AI project manager he both implements AI tools in clinical practice and performs research in the development of AI-based applications, in collaboration with the University of Tilburg. Dr. Ranschaert is visiting professor at the Ghent University and president of the European Society of Medical Imaging Informatics (EuSoMII). He is co-editor of *Artificial Intelligence in Medical Imaging* (Springer), co-author of three book chapters and of three scientific papers on the topic of AI in radiology. He was the invited speaker for many societies and scientific meetings in Europe and further abroad. He is Chief Medical Officer for the Diagnose.me platform and has functioned as advisor in several organizations and companies.

### Dr. René Van Tiggelen

**Figure:**
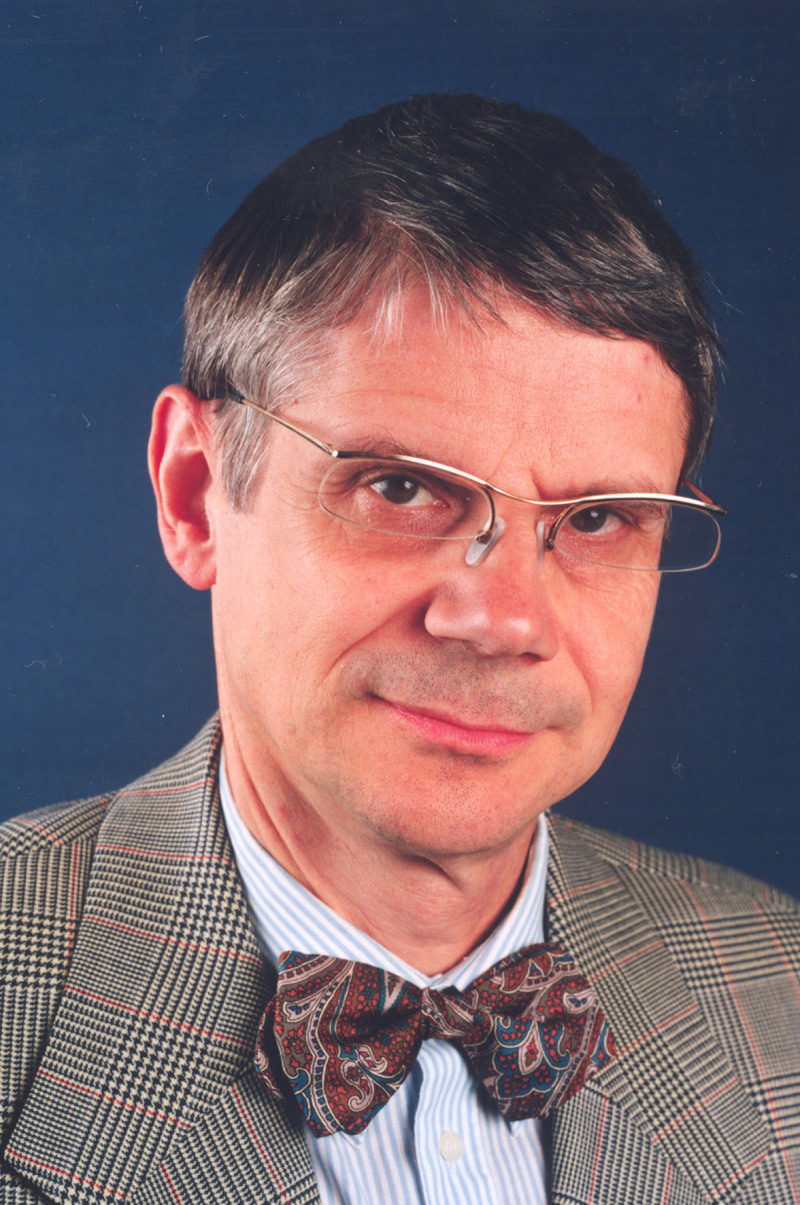


Dr. René Van Tiggelen graduated in Medicine at the University of Louvain (UCL-1967). He then decided to specialize in radiology under the leadership of Professors P. Bodart (UCL) and G. Cornélis (UCL/KUL) and simultaneously obtained a degree in social medicine and hospital management. He made his whole career as a radiologist in the Belgian armed forces. As an army medical officer with the rank of Colonel, he used to be the deputy chief of staff of the medical department. As a senior hospital lecturer he taught bone radiology at the VUB (Brussels Free University, Flemish section) from 1982 to 1996 and has been a guest teacher at the EHSAL since 1998. With a number of volunteers he created the Belgian Museum of Radiology in 1990 and has been its managing director since.

## Moderators

### Prof. Dr. Lieven Van Hoe

**Figure:**
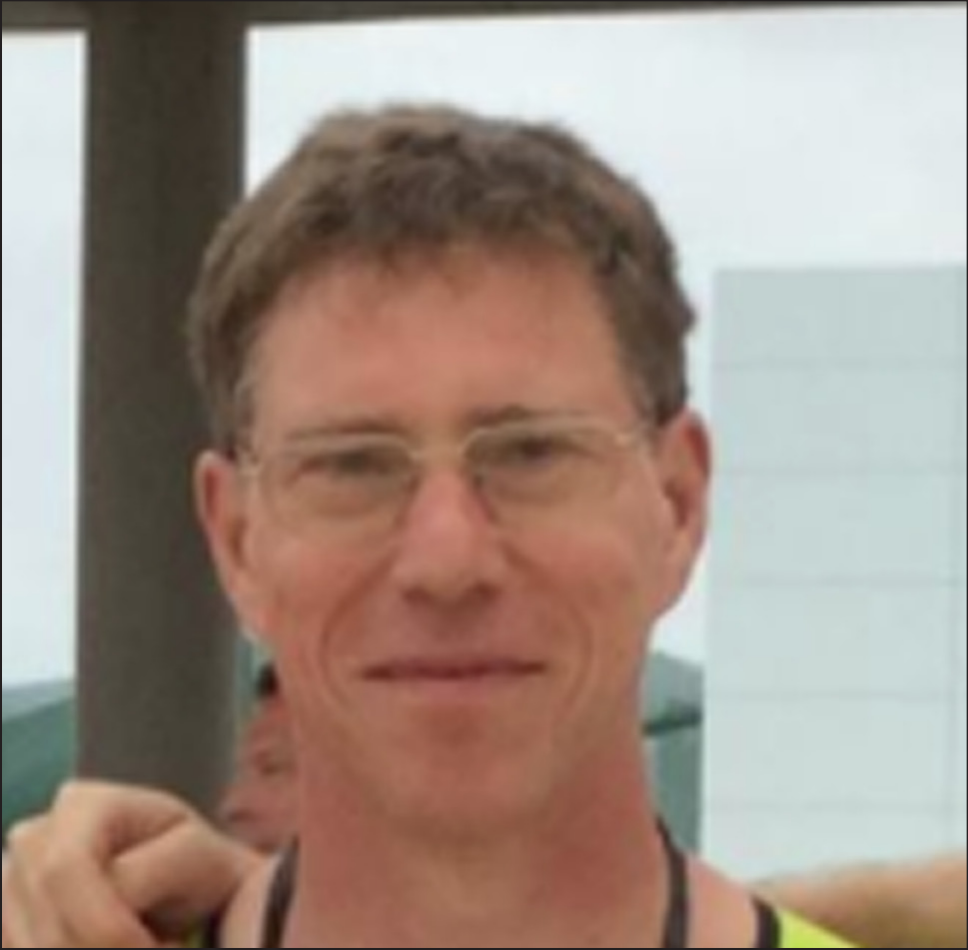


Prof. Dr. Lieven Van Hoe (MD, PhD) is a staff radiologist at OLV Hospitals Aalst-Asse-Ninove, with a PhD in fast volumetric data acquisition in abdominal CT and MRI. He is the current section president in abdominal imaging of the BSR and has a special interest in deep learning.

### Dr. Annemiek Snoeckx

**Figure:**
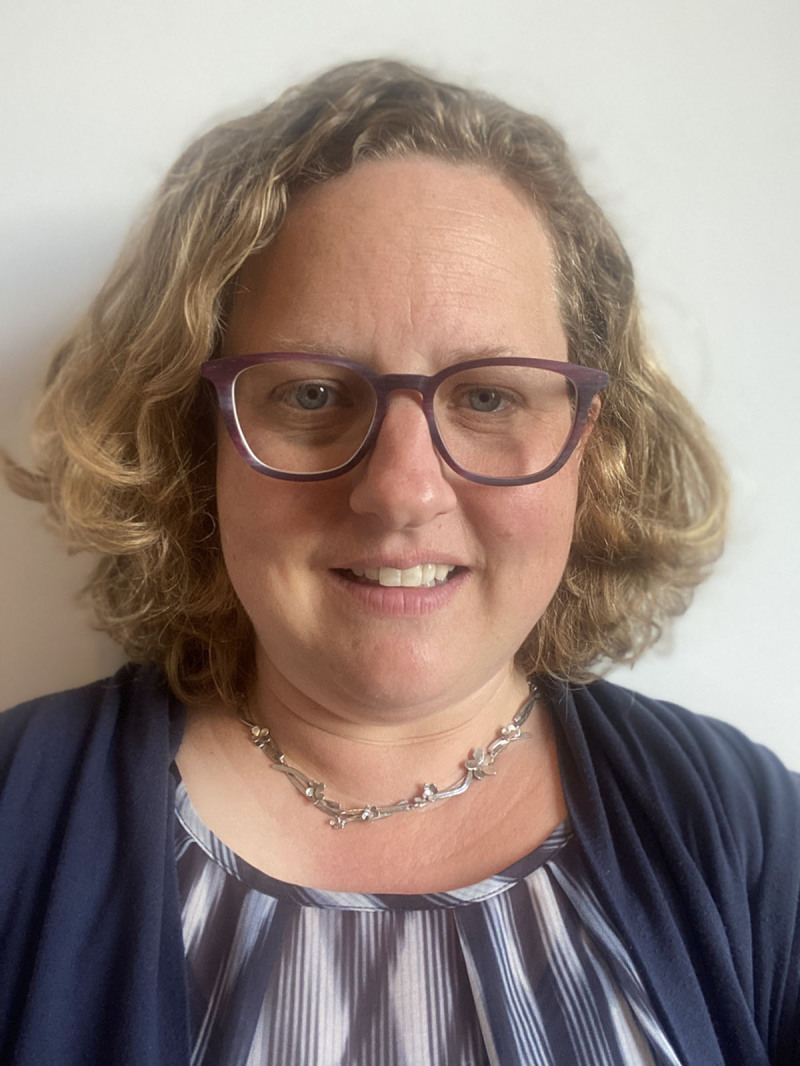


Dr. Annemiek Snoeckx (MD, PhD) is a radiologist at the University Hospital of Antwerp, Belgium. She graduated as Medical Doctor in 2003 at the University of Antwerp. She is a board-certified radiologist since 2008 and has been working since then at the University Hospital of Antwerp. Her main field of interest is chest imaging, with a focus on thoracic oncology imaging in general, pulmonary nodules, and lung cancer screening. She earned a PhD in 2019. She is an active member of the Belgian Society of Radiology (BSR), the European Society of Thoracic Imaging (ESTI), the European Society of Radiology (ESR), the European Respiratory Society (ERS) and the International Association for the Study of Lung Cancer (IASLC). She is the Dutch-speaking representative of the BSR chest section. On a European level, she is a member of the ESTI Training and Educational Committee, Diploma Committee and Lung Cancer Screening Certification Project. She is team leader of the Chest Radiology Written Evaluation Committee for the European Diploma in Radiology (EDIR). Her research interests are in the field of pulmonary nodules, artificial intelligence, lung cancer screening and Dual-Energy CT in lung cancer imaging. She authored more than 60 papers in peer-reviewed journals and eight book chapters. She has lectured at many national and international meetings and courses.

### Dr. Flavien Grandjean

**Figure:**
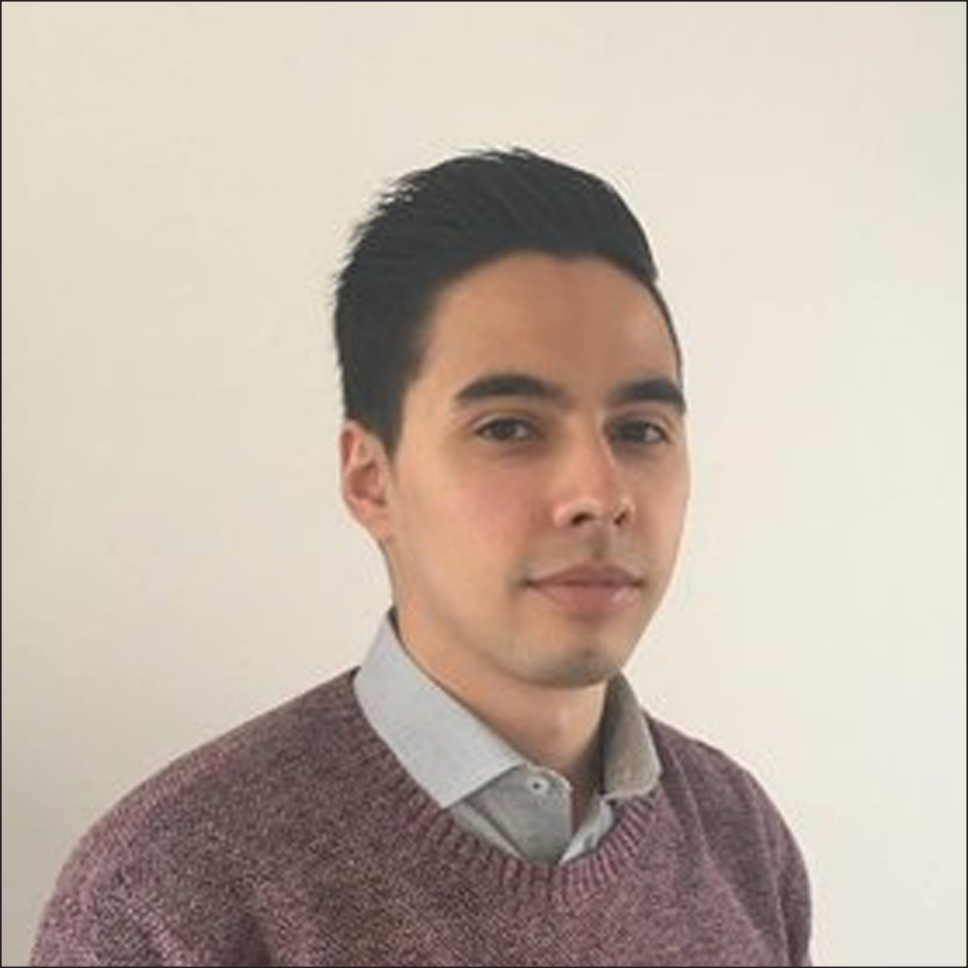


Dr. Flavien Grandjean is currently a third-year resident at the CHC Liège MontLegia and is the current French-speaking YRS president after being a YRS member since 2018 and vice-president in 2019–2020.

### Dr. Anne-Sophie Vanhoenacker

**Figure:**
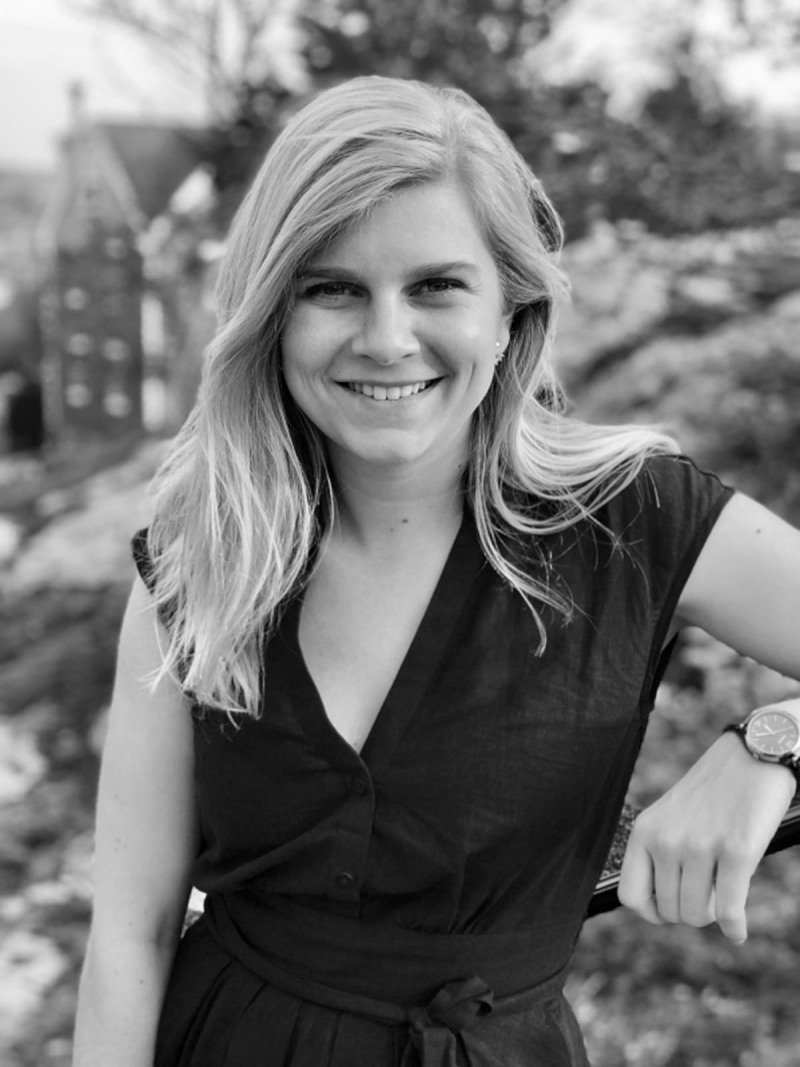


Dr. Anne-Sophie Vanhoenacker is currently a fifth-year resident at the University Hospitals of Leuven and is the current Dutch-speaking YRS president after being a YRS member since 2016, secretary in 2018–2019 and vice-president in 2019–2020.

## Biliary pathologies and interventional implications

Robert Ferdinand Dondelinger, MD, PhD

Department of Radiology, CHU Sart Tilman, Liège

The biliary system comprises intrahepatic ducts, main extrahepatic duct and gallbladder. As for any other anatomical ductal system, biliary pathologies translate basically either into luminal obstruction or wall leakage or an association of both. Focal duct narrowing leads to dilatation above the level of obstruction and jaundice. Bile leakage ends up into formation of biloma or biliary ascites. Cholangitis, abscess, or sepsis are frequent complications in both scenarios. Causes of bile duct obstruction are malignant or benign and can be schematically allocated to preferential levels of occurrence: cholangiocarcinoma or invasive gallbladder carcinoma are characteristic for hilar obstruction, impacted gallstones, pancreatic cancer, pancreatitis or papillary disorders obstruct the distal duct. Metastatic adenopathies occur predominantly at the level of porta hepatis, the rare Mirizzi syndrome at the confluence of the cystic duct and the main duct. Bile duct obstruction caused by benign inflammatory disease, postoperative strictures, and developmental bile duct abnormalities are other clinical settings. Cross-section imaging by CT or MRI, or centered imaging during ERCP, PTC, endoscopic US and biliary endoscopy give the clue to level and cause of obstruction in the vast majority of cases. At present, diagnostic tissue is best obtained by retrograde or antegrade endocanalar forceps biopsy or brush cytology. Bile leakage can occur at any level and is predominantly related to wall injury, infection, or ischemia, as it may occur in postoperative anastomotic strictures. The basic principle of treatment of bile duct obstruction resides in any form of temporary or permanent restoration of bile flow by catheters or stents, internal drainage being preferred. Leakage is treated by catheter evacuation of a collection of bile. Reaccumulation of biloma is prevented by diversion of bile from the site of leakage and bridging the site of injury, allowing for spontaneous wall healing. Since the introduction of the skinny 22-gauge Chiba needle in 1969, percutaneous fluoroscopically guided opacification of intrahepatic bile ducts became routine, carrying a minimum of significant clinical complications, provided punctured dilated bile ducts under elevated pressure, are drained during the same session. In practice over the years, ERCP and more recently, sophisticated EUS guided techniques have evolved as standard non operative methods for both, diagnostic and therapeutic approach of bile duct pathologies. Today, percutaneous intervention to the biliary system is relegated to situations, where a retrograde catheterization is not realistic or impossible for anatomical or technical reasons, which accounts roughly for about 10% of mixed cases mainly in invasive hilar biliary obstruction or prohibitive anatomical changes. A combined small-gauge percutaneous transhepatic and endoscopic rendez-vous manœuvre carries less trauma to the liver parenchyma. Self-expandable metal stents have become established standard solutions of bile flow restoration in malignant obstruction. Retrievable covered metal stents are advised for treatment of benign strictures. Percutaneous catheter drainage of acute, acalculous or calculous cholecystitis is helpful in critically ill patients as a temporizing measure. Occasional residual postcholecystectomy gallstones are removed through a T-tube tract. Intrahepatic gallstones are retrieved during repetitive sessions through a biliary-intestinal anastomosis.

## MR Rectum. How I Do It

Raphaëla Carmen Dresen, MD, PhD, Vincent Vandecaveye MD, PhD

Department of Radiology, University Hospitals Leuven

The aim of rectal cancer treatment is to provide local and distant control, in order to maximize potential for treatment success and survival. However, one treatment strategy does not fit all rectal cancer patients. The choice for optimal treatment has evolved in the last decades from standard surgery for all patients to a more individualized approach. This approach mainly depends on the risk assessment of disease recurrence. The most important risk factors are tumor and lymph node stage, distance to the anorectal junction and mesorectal fascia, and the presence of extramural venous invasion (EMVI). According to risk stratification, patients may receive surgery alone (local excision or total mesorectal excision [TME]), neoadjuvant chemoradiotherapy (in a standard or intensified scheme) followed by (extended) TME or local excision, or, in complete responders followed by a watch and wait strategy (which implies no surgery at all, but a strict follow-up program with the combination of digital rectal examination, endoscopy, and MRI). It is clear that accurate staging in this setting plays a key role in the decision-making process. While endorectal ultrasound can be very accurate in the evaluation of early-stage disease, it has to cope with disadvantages such as operator dependency, limited visibility of lymph nodes as well as limited use in cases of obstructive tumors. MRI, however, has become one of the leading imaging modalities because of its superior soft-tissue contrast, multiplanar imaging, and the possibility of functional evaluation. With the combination of high-resolution T2-weighted images and diffusion-weighted images, MRI allows accurate identification of risk factors, not only in the primary setting, but also during restaging and follow-up (Figure 1). The MRI findings should be stated in a structured report to provide the clinicians with all the information needed for an optimal treatment decision. Management of rectal cancer patients should be performed in a multidisciplinary manner, combining the expertise of all the different involved disciplines, in order to provide the patients with the highest chance of survival with the lowest morbidity.

**Figure 1:**
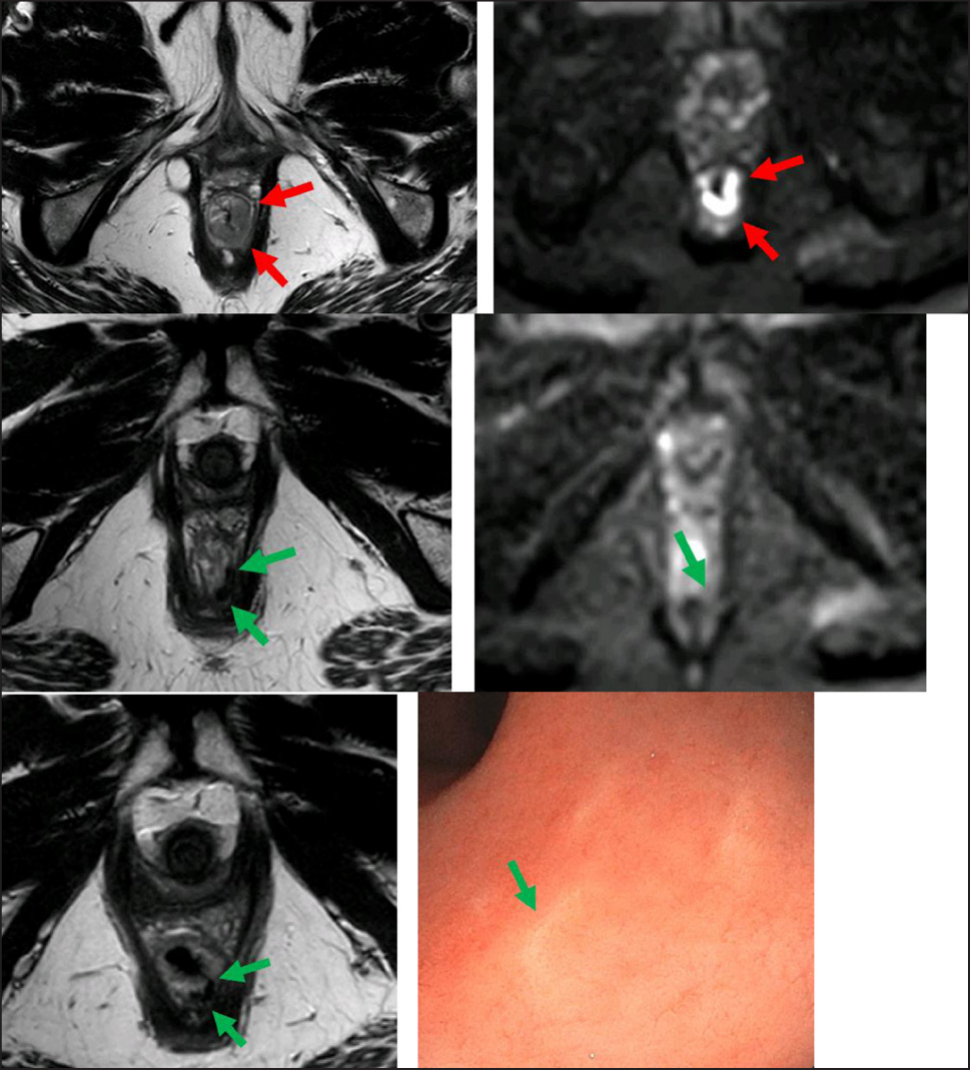
Patient with a tumor at the left posterolateral side of the distal rectum (see arrows on the images in the top row). After radiochemotherapy, the original site of the tumor has become very hypo-intense on the T2-weighted images with no restricted diffusion (see arrows on the ilages in the middle row), reflecting a complete response. This was confirmed by endoscopy, which showed a white scar (see arrow on the right bottom image).

The aims of this presentation are:

– To learn about the best imaging protocol for rectal cancer staging.
– To learn about risk factors and identify them on MRI.
– To learn how to interpret MR-images after (neoadjuvant) treatment.
– To learn how to identify clinical complete response.
– To learn how to make a structured MRI report.

## Adrenal Incidentaloma: How to Manage Them

Philip Van Hover, MD

Department of Radiology, OLV Aalst

Why adrenal imaging?

Because we have to know the difference between a malignant or benign lesion and if the lesion is secreting hormones. Which are the “dangerous” lesions, the “good” lesions and what are collision lesions. There are several techniques which we can use or on which these lesions are detected. What is the role of unenhanced CT, delayed CECT (contrast enhanced CT), in and out phase T1 imaging and one phase CECT? Also the role of PET/CT and dual source CT will be highlighted. At the end a clinical and radiological pathway will be given for daily practice.

## Abdominal Internal Herniations

Paul Meunier, MD, PhD

Department of Radiology, CHU Sart Tilman, Liège

This topic concerns a greater number of clinical situations than one would expect. In fact, internal hernias make up as many as 6% of occlusive syndromes. After a short embryological and anatomical reminder, the different types of hernias will be presented on the basis of their appearance via a normal or abnormal orifice. The different anatomical and vascular markers will be emphasized for each type of hernia in order to demystify the diagnostic reasoning. Finally, post-surgical iatrogenic hernias will be discussed.

## Imaging Beyond the Peak: Long-Term Pulmonary Changes after COVID-19

Walter De Wever, MD, PhD, Adriana Dubbeldam, MD, Johan Coolen, MD, PhD

Department of Radiology, University Hospitals Leuven

Many clinical studies have focused on the epidemiological, clinical, and radiological characteristics of inpatients with COVID-19 disease. There are only a few reports about the clinical follow-up of discharged patients.

Severe acute respiratory syndrome coronavirus 2 (SARS-CoV-2) is a highly contagious disease, and its first outbreak was reported in Wuhan, China. On January 30, 2020, the World Health Organization (WHO) declared it a pandemic disease. Chest CT examination has an important role in the evaluation of COVID-19 patients with false-negative RT-PCR results and studies reported the CT sensitivity as 98%. CT examinations also have great significance in monitoring disease progression and evaluating therapeutic efficacy.

Awulachew E. et al. demonstrated in his meta-analysis that common CT imaging features of COVID-19 pneumonia include frequent involvement of bilateral lung infections, ground-glass opacities (GGO), consolidation, crazy paving pattern, air bronchogram signs, and intralobular septal thickening. Bilateral involvement was common while single lobe involvement was rare. These signs of CT imaging might be an important tool for diagnosis and monitoring disease progression in patients with COVID-19 infection [1].

The extent of CT abnormalities progresses rapidly after the onset of symptoms, with a peak around days 6–11. The temporal changes of the diverse CT manifestations followed a specific pattern, which might indicate the progression and recovery of the illness.

At the moment of discharge, most of the patients have residual disease on chest CT scans. Ground-glass opacity is the predominant abnormality, followed by a mixed pattern (ground-glass opacity and consolidation). Pure ground-glass opacity is the most commonly seen subtype. Ground-glass opacity with irregular lines and interfaces are signs that can predict the development of interstitial fibrosis and needs long-term follow-up.

Liu et al. showed in their study that, compared with the abnormalities found on the last CT scans before discharge, the abnormalities in the lungs at the first (2 weeks) and second (4 weeks) follow-ups after discharge had been gradually absorbed. The lung lesions of 25.5% patients were shown to be fully absorbed on the first CT scans after discharge, and the rate of lung recovery increased to 64.7% after the second follow-up (Figure 1) [2].

**Figure 1:**
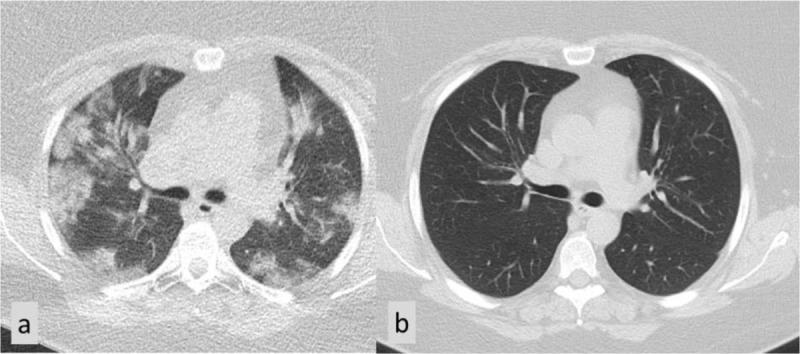
A 43-year-old woman showed on covid-scan multiple areas of ground-glass opacities in both lungs **(a)**. A control CT scan after 18 weeks could not show any abnormalities anymore **(b)**.

**Figure 2:**
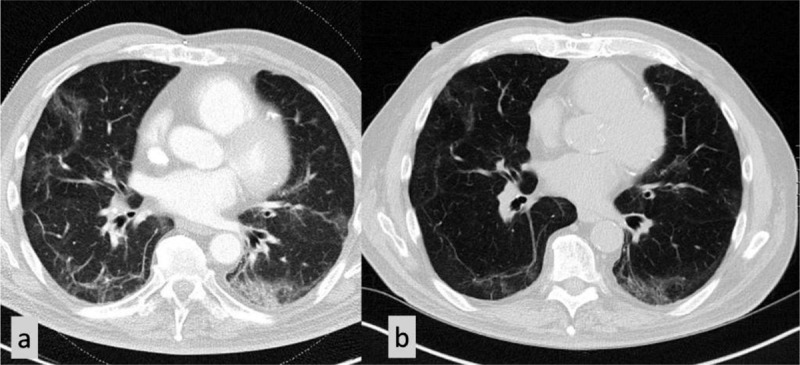
A 73-year-old man showed on covid-scan multiple areas of ground-glass opacities in both lungs but also a more reticular pattern with thickening of intralobular lines and irregular lines and bands **(a)**. A control CT scan after 20 weeks showed the persistence of irregular lines and discrete signs of interstitial fibrosis **(b)**.

**Figure 3:**
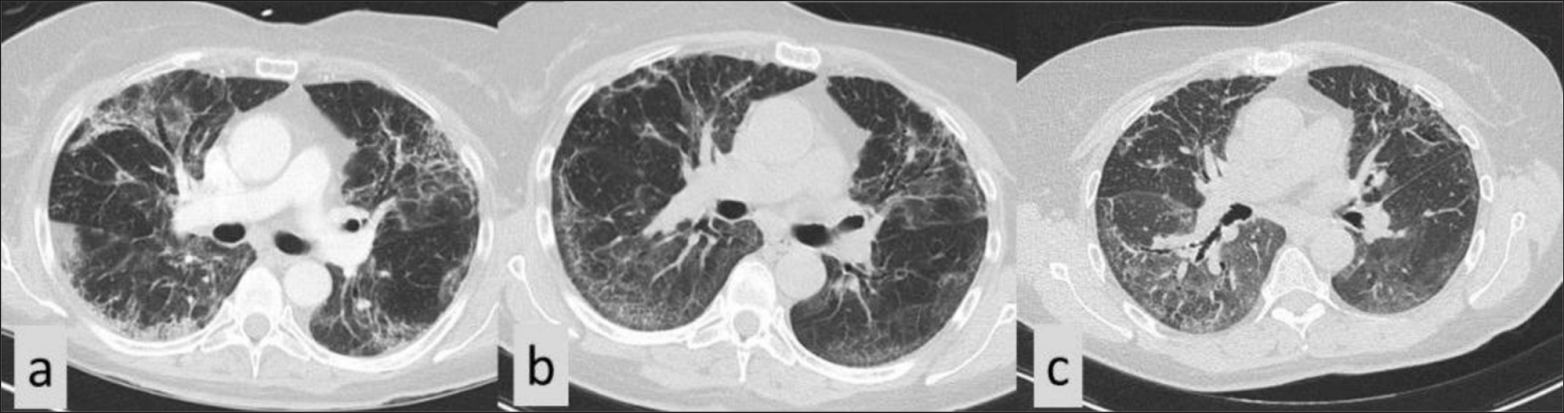
A 62-year-old woman showed on covid-scan multiple areas of peripheral ground-glass opacities in both lungs but also a more reticular pattern with interstitial thickening and irregular lines and bands **(a)**. A control CT scan after 20 weeks showed the persistence of irregular lines and signs of interstitial fibrosis **(b)**. A late control CT after 6 months showed the persistence of signs of interstitial fibrosis **(c)**.

Minhua Yu et al. found in their study that interstitial thickening, irregular interface, coarse reticular pattern, and parenchymal band, manifested in the process of the disease, may be predictors of the development of late pulmonary fibrosis. Irregular interface and parenchymal band could predict the formation of pulmonary fibrosis early (Figures 2 and 3) [3].

## Clinical Findings after COVID-19

Natalie Lorent, MD, PhD

Department of Pulmonology, University Hospitals Leuven

COVID-19, the disease caused by SARS-CoV-2 that has spread globally since the end of 2019, can present a wide spectrum of disease ranging from asymptomatic infections over mild to moderate respiratory symptoms to severe viral pneumonia with acute respiratory distress syndrome. The main target are the lungs but other organs, such as heart, kidneys, gut, can be affected in the different disease stages. Risk factors for severe disease are older age, arterial hypertension, cardiovascular disease, and chronic respiratory illness. Case fatality rates are difficult to assess based on the current data but could be as high as 1%, much higher than influenza, and this especially holds true for the elderly. Clinical outcomes of patients recovering from COVID-19 vary also by disease severity: in some cases it can result in serious lung damage with long-term consequences; however, it has been remarkable to note the recovery rate of patients following admission with moderate to severe COVID-19 pneumonia as for their clinical, radiological, and functional status. I will discuss the latest data on recovery after COVID-19 based on literature data as well as our own follow-up findings in UZ Leuven.

## COVID-19 beyond Lung Parenchymal Changes: Neurological Patterns

Sofie Van Cauter, MD, PhD

Department of Radiology, ZOL Genk

Various neurological and neuropsychiatric complications related to COVID-19 have been described, either during the acute phase, related to direct viral cytopathy or a para-infectious cytokine storm, as well as in later stages reflecting an immune or antibody-related inflammatory response. Although these complications are relatively rare, such patients are often severely affected, resulting in poor outcomes. In this talk, we will touch upon the mechanisms related to neuroinvasion and discuss COVID-19-related neurological disease.

## Artificial Intelligence in COVID-19

Laurens Topff, MD

Netherlands Cancer Institute (Amsterdam, Netherlands)

Erik Ranschaert, MD, PhD

ETZ Tilburg (Netherlands), University of Ghent (Belgium)

Soon after the coronavirus outbreak, it became clear that medical imaging has an important role to play in the fight against COVID-19. From a strong need of both hospitals and artificial intelligence (AI) companies to tackle this pandemic, collaborations were started with a speed that would be unthinkable outside times of crisis. It became clear that in such exceptional situations with an impact on the society as a whole, there is an enormous willingness to share data, infrastructure, and AI knowledge for a common purpose. The application of deep learning has the potential to optimize and expand the role of imaging in COVID-19. In many centers, CT and/or chest X-ray was used for triage and timely diagnosis of pulmonary infection. Starting from the idea that AI-tools have the potential to support the radiologist in accurately and timely reporting radiology examinations performed in patients suspected of COVID-19, several projects were launched to develop applications for the automated analysis and quantification of pulmonary infection. The first studies on Chinese data showed that there is a relationship between the extensiveness of abnormalities on imaging and the severity of disease. In addition, it is being investigated how imaging can predict clinical evolution, such as the need for admission to intensive care or the need for ventilation. Such AI applications can play a role in future disease outbreaks. Not only in China, but also in other countries such as Belgium and the Netherlands, several initiatives to develop algorithms related to COVID-19 and radiology emerged in no time. In this presentation an overview will be provided of the different initiatives that have been undertaken in the field of AI, with a special focus on Europe. Not only the radiological and scientific, but also the technical and medical-legal aspects will be explained.

## Movie: Film: Röntgen’s X-rays (Re)Discovered

René Van Tiggelen, MD

Curator Belgian Museum for Radiology, Military Hospital, BRUSSELS

Professor Roentgen’s last will determined that all his research notes were to be burnt after his death. This is the reason why we thought it useful to make a film reconstituting how the most important discovery of the 19th century was probably made. In this movie (13’) the physical properties of X-rays are also explained.
